# Challenges and Future Recommendations for Lightning Strike Damage Assessments of Composites: Laboratory Testing and Predictive Modeling

**DOI:** 10.3390/ma17030744

**Published:** 2024-02-04

**Authors:** Yeqing Wang, Yin Fan, Olesya I. Zhupanska

**Affiliations:** 1 Department of Mechanical and Aerospace Engineering, Syracuse University, Syracuse, NY 13244, USA; 2 School of Aeronautics and Astronautics, Shanghai Jiao Tong University, Shanghai 200240, China; 3 Department of Aerospace and Mechanical Engineering, University of Arizona, Tucson, AZ 85721, USA; oiz@email.arizona.edu

**Keywords:** composite materials, lightning strike, laboratory testing, predictive modeling, material degradation

## Abstract

Lightning strike events pose significant challenges to the structural integrity and performance of composite materials, particularly in aerospace, wind turbine blade, and infrastructure applications. Through a meticulous examination of the state-of-the-art methodologies of laboratory testing and damage predictive modeling, this review elucidates the role of simulated lightning strike tests in providing inputs required for damage modeling and experimental data for model validations. In addition, this review provides a holistic understanding of what is there, what are current issues, and what is still missing in both lightning strike testing and modeling to enable a robust and high-fidelity predictive capability, and challenges and future recommendations are also presented. The insights gleaned from this review are poised to catalyze advancements in the safety, reliability, and durability of composite materials under lightning strike conditions, as well as to facilitate the development of innovative lightning damage mitigation strategies.

## 1. Introduction

Composite materials play a prominent role in the design of high-performance structures capable of withstanding extreme structural loads [1]. Polymer matrix composites revolutionized the structural design of aerospace structures due to their light weight, high stiffness, and high strength. At the same time, the survivability of polymer matrix composites subjected to harsh environmental conditions is still of concern. Lightning strikes [2] are high-risk and low-probability (e.g., one and a half strikes per year per airplane) events that present significant threats to polymer matrix composite structures. Carbon fiber–polymer matrix composites (CFRP) are particularly vulnerable to high-energy lightning strike events due to their relatively low electrical and thermal conductivities as well as limited service temperature and significant degradation in properties above the decomposition temperature. The combined effect of these factors is manifested in the lightning-induced heat damage zone. The damage induced by a lightning strike is difficult to prevent [3], though protections, such as copper foils, have been applied [1]. By accurately predicting the behavior of composite materials under lightning strikes, it is possible to enhance the design and safety of various structures and components.

Accurate models for simulating lightning strike on composites require a comprehensive approach that considers various factors that reflect the damage nature of a lightning strike on composite materials. This includes, first of all, electro–thermal–mechanical coupling, which involves accounting for the intricate interactions between electrical, thermal, and mechanical phenomena within the composite material during a lightning strike, requiring sophisticated multi-physics simulations. Detailed information on the material’s electrical, thermal, and mechanical properties, particularly with consideration for temperature-dependent behavior is another critical aspect that high-fidelity models need to address. Establishing models that predict temperature-dependent properties can significantly enhance the accuracy of simulations and better capture the dynamic response of composites during lightning strike events. In addition to thermal damage, mechanical damage caused by thermal stress and shockwave pressure [4,5,6] deserves attention. Effective damage prediction methods are essential for assessing the potential effects of lightning strikes on composite materials. These methods need to consider the damage characteristics of composite materials, including thermal decomposition of the matrix, delamination, matrix cracking, and fiber breakage, under the high-energy conditions of a lightning strike [7]. Lastly, the assessment model of residual mechanical properties, mainly for the strength of composite materials under lightning strike damages are required [8]. 

Laboratory lightning strike tests are crucial for providing empirical data to validate and improve predictive models. These tests aid in understanding the behavior of composites under lightning conditions, enabling the development of accurate material models and simulation parameters. The validation of predictive models is a key step toward ensuring their reliability. The integration of experimental data from laboratory tests, as well as in-service monitoring and inspection data, is crucial to validating the accuracy of the models and build confidence in their predictive capabilities. The extent of the damage obtained via optical analyses, C-scans and X-rays, is frequently used for the verification and validation of numerical simulation models for describing the damage induced in composite materials by an artificial lightning strike [3]. 

Existing research efforts include the development of advanced numerical techniques, such as finite element analysis and computational fluid dynamics, to simulate the complex phenomenon of lightning strikes on composite materials. Furthermore, experimental data are being utilized to validate and improve the accuracy of predictive models. Actually, even the state-of-the-art models of lightning strikes cannot consider all the factors above. This is because the multi-phase interaction between lightning and composites is not very clear until now and integrating realistic material properties into the models makes the solutions of multi-physic field equations difficult to obtain due to numerical convergence issues. For instance, most numerical analyses are conducted under the assumption that the electrical conductivities of the composite materials depend only on the temperature or degree of pyrolysis, to simplify the complication of lightning strike which is far away from the actual physical and chemical phenomena. To overcome this, an attempt of representative volume element (RVE) [9] containing fiber, resin, and a fiber–resin interface, was proposed to estimate the effective electrical conduction of CFRP composites. 

Looking ahead, future efforts should aim to enhance the fidelity of predictive models by incorporating more realistic and accurate material properties, considering the effects of environmental factors, and accounting for stochastic uncertainties in the simulation. Additionally, the integration of machine learning and artificial intelligence to optimize predictive models represents a promising avenue for future research.

## 2. Laboratory Lightning Strike Tests

Actual lightning strike discharges in nature are highly uncertain and extremely difficult to control for experimental implementation and instrumentation. Thus, simulated lightning strike tests are normally conducted in the laboratory condition for the following three purposes: (i) the observation and identification of the physics and failure mechanisms involved in the lightning strike interaction with materials and structures, (ii) the assessment of the damage tolerance of composite materials against a lightning strike, and (iii) the evaluation of the effectiveness and performance of conventional and innovative lightning strike protection solutions. Depending on whether the testing material or structure is electrically conductive or non-conductive, laboratory lightning strike tests are often classified into two categories: (i) high-current tests and (ii) high-voltage tests. If the material is electrically conductive, such as CFRP composites (e.g., for aircraft fuselage), high-current (up to 200 kA) lightning strike tests should be performed since the thermal damage caused by the conduction of the lightning strike current represents the primary damage. Whereas if it is electrically non-conductive, such as glass fiber-reinforced polymer (GFRP) composites (e.g., for wind turbine blades), high-voltage (1000 kV/µs) lightning strike tests should be performed as the puncture damage due to the dielectric breakdown is the primary damage [10]. Note that high currents and high voltages often cannot be simultaneously generated in the testing facility due to the current limitations of the power rating of the capacitors. The methods of both high-current tests and high-voltage tests are detailed in the prevalent lightning strike testing standards, such as the SAE ARP-5412 [11].

**Figure 1:**
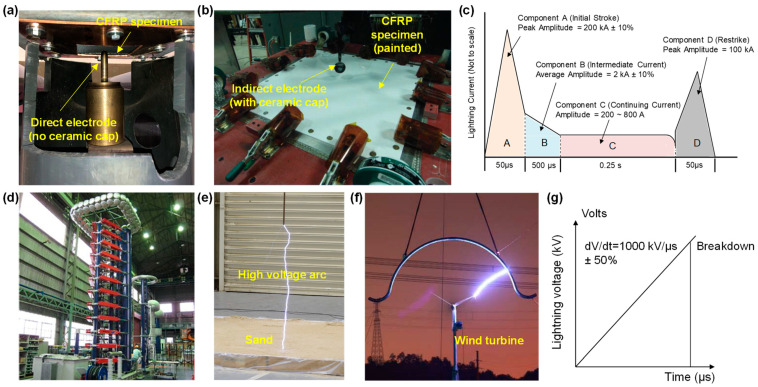
Simulated lightning strike tests: (**a**) high-current test setup with a direct electrode at the High Voltage Lab at Mississippi State University (MSU-HVL) [12], (**b**) high-current test setup with an indirect electrode at the National Technical Systems in Pittsfield, Massachusetts [15], (**c**) standard lightning high-current test waveform [11], (**d**) Marx high-voltage generator produced by Haefely test AG in Japan [16], (**e**) high-voltage lightning test on sand at MSU-HVL [17] (**f**) high-voltage lightning test on a wind turbine in China [18], (**g**) standard lightning high-voltage test waveform A suggested by the SAE 5412 standard [11].

High-current lightning strike tests for CFRP composites typically use a cathode–anode configuration to produce a high-intensity electric arc in the air. Figure 1 shows examples of the testing setups [12]. In the U.S., such a testing capability is available at the National Institute of Aviation Research at Wichita State University [13], High Voltage Lab at Mississippi State University (MSU-HVL) [14] (Figure 1a), and the National Technical Systems in Pittsfield, MA, USA [15] (Figure 1b), etc. Depending on the polarity of the electrode used in the test, the CFRP composite specimen can either serve as a cathode or an anode. The produced electric arc attaches to the surface of the CFRP composite specimen, thereby simulating the lightning strike interaction with the CFRP composites. The actual lightning strike discharge contains multiple strokes with different durations and magnitudes of the electric current. This is simulated by a standard four-component current waveform (see Figure 1c), which is widely known to this research community. High-voltage lightning strike tests are typically for electrically non-conductive materials, such as glass fiber-reinforced–polymer matrix composites, which are commonly used for radome for aircraft and wind turbine blades. Figure 1d shows a Marx high-voltage generator produced by Haefely test AG in Japan [16], and Figure 1e shows a high-voltage lightning test conducted on sand at MSU-HVL [17]. Figure 1f shows a high-voltage lightning impulse test on a wind turbine conducted by a research group of Wuhan University in China [18]. The lightning voltage waveform used in the tests is suggested also by the SAE ARP-5412 [11] and is shown in Figure 1g. In the following discussions, we focus on the review of high-current lightning strike tests for CFRP composites.

Generating the four current waveforms (see Figure 1c) sequentially in the laboratory condition is still challenging due to the limitations of experimental capabilities. Most research papers [19,20,21,22,23,24,25,26,27] related to the lightning strike testing of CFRP composites recognized that the electric currents of waveforms B, C, and D are much lower than that of waveform A and assumed that the waveform A current contributes to the majority of the damage in the CFRP composites. Thus, these studies conducted only simulated lightning strike tests (and simulations) with a waveform A current. With the recent development of testing capabilities, a limited number of recent experimental studies have produced multiple and sequential waveforms (e.g., waveforms C and D, waveforms A, B, and C) [28,29,30]. More recently, one notable study successfully produced electric arcs with a complete sequence of all four waveforms [31]. These studies with combined waveforms demonstrated that considering only the waveform A significantly underestimates the lightning strike damage in the composites. For instance, a single waveform A current can cause a damage depth of 0.513 mm and a damage area of 1104 mm^2^ in a CFRP composite specimen, whereas combined waveforms A, B, C, and D result in a damage depth of 1.280 mm and a damage area of 2790 mm^2^ [31]. Therefore, we would like to point out that conducting only waveform A tests could be sufficient for some comparison studies (e.g., comparing the lightning strike damage in CFRP composites with and without lightning strike protections). However, it is clearly insufficient if the purpose of the experimental study is to understand the damage mechanisms or assess the damage tolerance of the CFRP composites (especially for unprotected CFRP composites) in actual lightning strike conditions. The underestimated damage results from waveform A tests will lead to a less conservative design of CFRP composite structures and hence may lead to risks of structural failure during operation and a reduced structural lifetime.

In the paragraphs below, our review will attempt to answer the following four questions: (i) do laboratory lightning strike tests really represent natural lightning strike? (ii) what information is needed for lightning strike modeling in experimental testing? (iii) what is the role of lightning strike testing in model validation, and (iv) what is the relevance to composite structures?

To answer the first question, the characteristics of laboratory electric arcs and actual lightning strikes are reviewed and compared in Table 1. It can be seen that the laboratory electric arc and the actual lightning strike share similarities in the context of peak current, peak power, action integral, and discharge mechanisms. However, differences still exist in the levels of electric voltage and acoustic shock wave and the environments between the laboratory condition and the actual flight/operation conditions (e.g., altitude, aircraft movement, and temperature and humidity). These differences may lead to the underestimation of the lightning strike damage in the CFRP composites. For example, the voltage of the laboratory lightning strike test is at least 1000 times lower than the actual lightning strike voltage. Such a reduction in the voltage will potentially lead to the underestimation of the dielectric breakdown damage. The moisture will lead to reductions in dielectric breakdown strength and hence make the composites more prone to dielectric breakdown damage [32,33]. For instance, experimental results indicate that Cyanate Ester/S2 glass composite retains 90% of its dielectric strength after six months of exposure to 99% humidity [33]. Thus, there is a need to further innovate testing techniques to simulate the actual lightning strike condition in the laboratory conditions with a higher degree of fidelity. 

The second question is related to the information needed for the lightning strike modeling of CFRP composites in laboratory lightning strike tests. To answer this question, we discuss it from two aspects: (i) the loading conditions from the electric arc and (ii) the initial and boundary conditions of the CFRP composites. For the first aspect, the determination of the loading conditions still heavily relies on assumptions or calculations without proper validations. Researchers initially applied high-intensity electric arcs as a concentrated electric current load at a specific node in finite element models [19,23,35]. Then, it was found that doing so would underestimate the damage because it ignored the expansion of the electric arc in the radial direction. For example, it has been reported that ignoring the arc expansion has led to an underprediction of damage of 56% [16,36]. A recent study [6] shows that the arc channel expansion morphology is dominated by the outermost fiber orientation.

Subsequent modeling works have assumed a uniformly or non-uniformly (e.g., Gaussian-shaped) distributed current density and heat flux with a fixed or expanding radius to model the lightning strike loading condition [22,37,38,39,40,41], which have significantly improved the model fidelity. More recently, researchers have developed plasma models to predict the heat flux and the current density in the electric arc and the arc expansion using the classical magnetohydrodynamic (MHD) method [42,43,44,45,46]. Despite significant progress being been made on the modeling of the lightning strike loading conditions, experimental data are still lacking to verify the assumed loading conditions or validate the predictions from the MHD plasma model. Existing experimental data on the plasma temperature available in the literature [47,48,49] are for electric arcs for welding applications. Such data cannot be used to validate the lightning strike loading conditions since the electric current for welding applications is much lower than the electric current of the lightning strike discharge (i.e., 100–800 A for welding vs. 200 kA for lightning strike impulse current). Therefore, there is a need to develop experimental measurement and instrumentation techniques to characterize the high-intensity lightning strike electric arc and provide data to validate the loading conditions used in lightning strike modeling.

**Figure 2:**
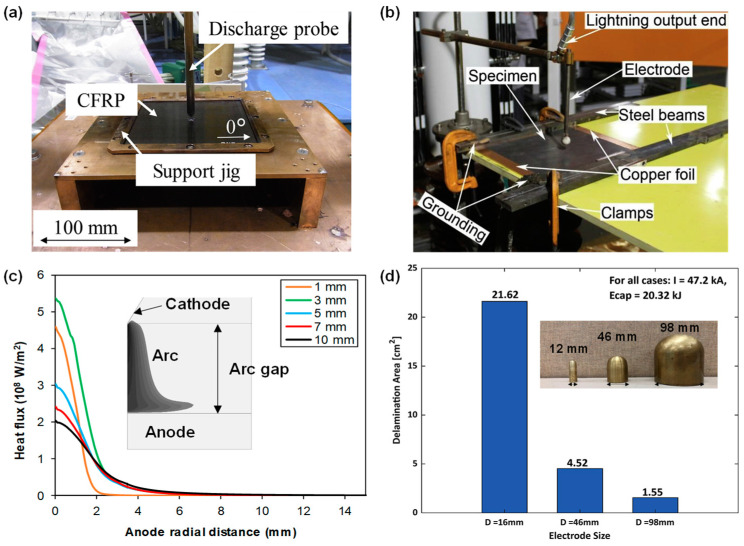
Electrode setups used in simulated lightning strike tests: (**a**) an example of a direct (needle) electrode (without insulating cap) [50], (**b**) an example indirect (jet diverter) electrode (with insulating cap) [30], (**c**) simulation results showing the effect of the arc gap on the predicted heat flux within an electric arc [51], (**d**) experimental results of the delamination area in CFRP composite laminates caused by using different sizes of direct electrodes in the simulated lightning tests [12].

Note that various factors involved in the lightning strike tests can affect the characteristics of the electric arc, and hence also the lightning strike load on the CFRP composites, such as the distance between the electrode and the surface of the CFRP composite laminate (or the “arc gap”), the electrode shape and size, direct vs. indirect electrode (see Figure 2a,b), and the grounding configurations [12,50,51,52]. A simulation study has shown that the heat flux of the electric arc decreased by 80% as the arc gap increased from 1 mm to 10 mm (see Figure 2c) [51]. The effects of the electrode shape and size and the grounding configuration on the lightning strike loading and the resulting damage in CFRP are discussed systematically in the literature with modeling and experimental testing (see Figure 2d [12]). Notably, the delamination area in the CFRP composite laminate caused by a lightning impulse current of 47.2 kA reduced from 21.62 to 4.52 and 1.55 cm^2^ as the electrode sizes increased from 12 to 48 and 96 mm in diameter. Interested readers are referred to these studies [12,51,52,53,54,55] for more details. For the second aspect, the information needed for establishing the initial and boundary conditions for the lightning strike modeling of CFRP composites include the geometry, the layup of the composite laminate, the grounding configuration, and the ambient temperature. For the lightning strike electric–thermal model, the ambient temperature will be applied as the initial boundary condition. A zero-electric potential will be applied on the sides where grounding is applied. Moreover, a surface radiation boundary condition needs to be applied to account for the radiative heat exchange between the external surfaces of the CFRP composite specimen and the ambient environment. A convective heat boundary condition may not be necessary due to the extremely short duration of the lightning strike [19,56]. For models that couple the electric–thermal response with the mechanical response, additional displacement boundary conditions need to be applied, which depends on whether the four edges are clamped or simply supported or having a mix of both [57,58,59,60,61]. The back surface velocity correlates with the mechanical impulse induced by the lightning strike and the inertia of the plate [3].

The third question is related to the role of testing in model validations. Numerous lightning strike damage models have been developed in recent years. One of the first models is the electric–thermal coupling model developed by Ogasawara et al. in 2010 [19]. Many of the subsequent lightning strike models were derived based on this model by including additional physics (e.g., mechanical pressure loading [22,60,61]), more accurate loading boundary conditions (e.g., from point electric current load to surface electric current load [22,37,38,39,40,41]), more accurate representations of the pyrolytic behaviors of the epoxy resin [39,62,63], and more sophisticated progressive material modeling techniques (e.g., element deletion [23,39,41]). These models are capable of predicting the extents of the delamination area and the material loss caused by the resin vaporization and the carbon fiber sublimation. Some other models can also predict the mechanical responses due to the lightning shock wave or the pressure load from the plasma and the electromagnetic force [57,60,61,64,65] and due to the thermal expansion. To validate the accuracy of these models, predictions from the models must be compared with experimental test results. Specifically, experimental results used in the model validations include the extent of the depth and area of the delamination and material loss characterized from ultrasonic inspections and/or destructive sectioning. Moreover, a few researchers have developed models to predict the compression-after-impact (CAI) response to study the residual strength and stiffness after the lightning strike impact [8,20,66,67] and have validated their models against the CAI test results. 

Overall, validations of the existing lightning strike models mostly rely on comparing predictions of the damage patterns/characteristics with those obtained after the simulated lightning strike tests. The time-dependent transient responses of the composite specimen subjected to the lightning strike impact (e.g., temperature and displacement histories, damage evolution) are often ignored. Although experimental test data on the temperature (backside temperature) and deflection histories of the composite specimen are available, these data are rarely used for model validations. Therefore, there is a need to validate lightning strike models by not only comparing predictions against experimental test data after the lightning strike impact, but also comparing predictions against transient responses during the lightning strike impact. This will ensure that the damage mechanisms, as well as energy dissipation mechanisms, are accurately captured using the developed models. Future experimenters are suggested to innovate the instrumentations to capture the damage evolution during simulated lightning strike tests.

The last question is related to the relevance of simulated lightning strike tests to composite structures. A lightning strike causes local damage to various types of composite structures, such as protected CFRP composites with expanded copper mesh, unprotected CFRP composites, composites with and without paint layers [24,29,30,68,69,70,71,72,73,74], composites with vertically interleaved fibers [74], composites containing electrically conductive nanofillers [75,76,77,78] and single-walled CNT tuball paper [79], composites with conductive coatings [80,81], metal-tufted composites [82], thermoset and thermoplastic composites [50,83], sandwiched composites [84], stitched composites [85], scarf-repaired composites [2], composites with mechanical fasteners [27], and adhesively bonded composite [86], as well as full-scale composite structures, such as wind turbine blades [18,87,88,89]. Although existing simulated lightning strike experimental studies for CFRP composites primarily focused on unprotected and protected composites and composites with mechanical fasteners, the other composite structures are also of significant importance. For example, it has been reported that the lightning strike damage mechanisms in painted and unpainted composite specimens are significantly different [24,64,73]. The paint layer tends to exacerbate the lightning strike damage. Moreover, the wreckage examination of Schleicher ASK 21 destroyed in a lightning strike accident revealed that bonded joints within the composite wings and in the fuselage were broken along the bond lines leading to the airframe disintegration [90]. Therefore, there is a need to investigate the lightning strike damage tolerance of other important composite structures (e.g., adhesively bonded composites, sandwiched composites) to expand the material database, providing insights into the different damage mechanisms and sufficient guidance to inform the design process.

## 3. In Situ and Post-Strike Characterization and Imaging

To understand the material behavior and damage mechanisms of the CFRP composites subjected to lightning strikes, in situ and post-strike characterization and imaging are effective tools. The in situ method is challenging due to the extremely short duration of the lightning strike, whereas the post-strike method is relatively more achievable and most widely used in the current lightning strike experimental studies. Below, we present a review on the in situ method first followed by a review on the post-strike method.

**Figure 3:**
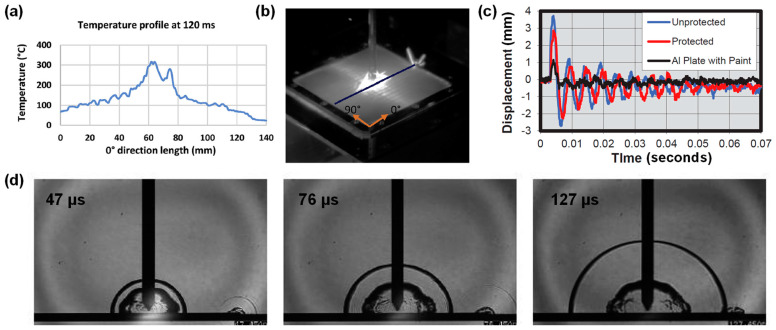
In situ characterization and imaging in simulated lightning strike tests: (**a**) temperature profile on the surface of a composite specimen measured using a thermal imager [91], (**b**) thermography image showing the electric current conduction path on the composite surface [91], (**c**) displacement of the composite specimen during simulated lightning strike [15], and (**d**) evolution of the arc and the shock wave generated during the lightning strike test using the shadowgraph imaging technique [92].

In situ characterization and imaging are crucial for understanding the time-varying responses of the CFRP composites during a lightning strike. For example, HD thermal cameras have been used to visualize the flow of the lightning electric current on the surface of a CFRP composite [6,15,91] and record the surface temperature history (see Figure 3a,b). Kumar et al. [91] used a thermal camera and captured the directional preference, which shows that the electric current flows mostly along the fiber direction (see Figure 3b). Moreover, high-speed digital image correlation systems and optical laser measurement sensors have been used to record the displacement history of the CFRP composite panels during lightning strike tests [15], which discovered the strong oscillations of displacement during lightning strike impact (see Figure 3c) and that the amplitude of the oscillation changes with different surface conditions (i.e., protected, unprotected, and with aluminum paint) (see Figure 3c). Furthermore, researchers have used the shadowgraph technique in lightning strike tests, which enabled them to visualize the shockwave propagation and the high-temperature gas generation due to the vaporization of resin and sublimation of carbon fiber [92], as shown in Figure 3d. The technique allowed them to study the effects of indirect vs. direct electrodes on the shockwave characteristics and resulting damage in CFRP composites. They found that shock waves generated by the electric discharge played a relatively insignificant role in damaging the CFRP composite. 

The post-strike characterization and imaging can be classified into two categories: non-destructive and destructive methods. The non-destructive method includes taking HD camera photos and using ultrasonic inspections and X-ray tomography. On the other hand, the destructive method includes fractography using scanning electron microscopy (SEM), optical microscopy, destructive sectioning, and residual strength tests using three-point or four-point flexural tests or compression-after-impact (CAI) tests. One recent study systematically pointed out the modifications necessary to standard CAI specimen geometry and test setup design to inflict specimen failure at the lightning damage site [93]. The ultrasonic inspection technique provides a projected area and position of the delamination in the CFRP composites. Specifically, a C-scan can be used to identify the extent of the delamination in the in-plane direction, while a B-scan provides the information of the depth of the delamination across the thickness of the composite. ImageJ software can be used to process the images obtained from the ultrasonic inspection and estimate the delamination area and depth. The fractography using microscopic imaging and destructive sectioning allows us to see the detailed microstructure at the lightning damage site and identify the different failure modes (i.e., fiber breakage and pullout, charring, matrix cracking, fiber–matrix debonding, delamination) and quantify the extent or number density of each failure mode. For example, Kumar et al. [94] used SEM and discovered a unique fiber damage morphology that could be highly pertaining to the internal arcing between adjacent fibers in CFRP composites. Residual strength tests allow us to check how much stiffness and strength of the CFRP composites have lost after the lightning strike tests. For example, it was reported that the compression-after-impact (CAI) strength experienced a reduction of 4.7%, 11.9%, and 32.4% for CFRP specimens subjected to 30, 50, and 70 kA of simulated lightning impulse current, respectively, in comparison with the reference specimen, which was not subjected to simulated lightning strike tests.

As discussed above, both in situ and post-strike characterization and imaging are extremely helpful for us to understand the lightning strike material behavior and failure mechanisms of the CFRP composites. For the in situ method, it is still challenging, primarily owing to the strict requirements on the instruments, which must be compatible with the high voltage environment, have sufficient high resolution to acquire data within microseconds, and be able to filter the excessive brightness during the lightning strike. Future research is recommended to improve the resolution and precision of in situ characterization and imaging, such that more in situ time-varying data can be acquired to gain deeper insights into the lightning strike interaction mechanisms with composites and provide more experimental data for model validations.

## 4. Material Characterization and Modeling

The behavior of composite materials under lightning strike conditions is intricately influenced by temperature-dependent properties. Temperature-dependent properties play a pivotal role in capturing the dynamic response of composites subjected to lightning strike events. The relevant material properties include electrical, thermal, mechanical, and viscoelastic characteristics, which undergo significant variations as the temperature changes during a lightning strike. Electrical properties, such as conductivity and permittivity, are inherently temperature-dependent and can markedly influence the flow of current within the composite material during a lightning strike. Accurately modeling these properties at different temperatures is essential for predicting the electrical behavior of composites under varying environmental conditions. There is a wide variation in the electrical properties at the room and high temperatures reported in the literature. Table 2 shows the prevalent anisotropic electrical conductivities for the unidirectional composites at room temperature used in studies on lightning strikes. The type of composite material is shown in parentheses after the first author’s name in the first column of the table.

Note that electrical conductivities reported in [19,22,95] were obtained experimentally. Studies [21,23] did not report how the electrical conductivities were determined. As for the temperature-dependent electrical properties, various assumptions were made. In [19], the electrical conductivity in the through-the-thickness direction of the CFRP is assumed to increase linearly from 7.94 × 10^−7^ S/m to 0.1 S/m (1.3 × 10^5^ times) when lightning-induced surface recession starts to occur at a temperature above 600 °C until the carbon fiber sublimation temperature at around 3000 °C. In [21], the electrical conductivities in the transverse direction and in the through-the-thickness direction of the CFRP composites are assumed to increase linearly from 0.001145 S/m to 2 S/m and from 3.876 × 10^−6^ S/m to 2 S/m, respectively, when the temperature exceeds 343 °C (when resin starts to decompose) until 500 °C; above that, the electrical conductivities are assumed to be temperature-independent. In [23], temperature-independent electrical conductivity was used. In [22], temperature-independent electrical conductivity for the CFRP composite was used, except for the electrical conductivity in the through-the-thickness direction, which was assumed to be five times higher when the temperature exceeds 600 °C. 

As for carbon fibers, their temperature dependence was found to be similar to that of semiconductors [96]. Typically, the electric conduction of a semiconductor is governed by lattice scattering when carrier concentration is intrinsic, particularly at high temperatures (>100 °C). The temperature-dependency when T > 100 °C can be characterized using the Arrhenius law. Sauder et al. [96] studied experimentally the electrical conductivity of a PAN-based fiber in the longitudinal direction at a temperature range 90~1800 °C. The activation energy reported for the temperature range 25~330 °C was Δ*E* = 0.0024 eV. At the temperature range 330~1800 °C, the activation energy was Δ*E* = 0.12 eV, and this value was assumed for the temperature range 1800~3316 °C.

Thermal properties, including thermal conductivity and heat capacity, influence the dissipation and propagation of heat generated during a lightning strike. Modeling the temperature-dependent thermal properties of composites is crucial for simulating the thermal response and potential damage mechanisms, aiding in the evaluation of the material’s thermal performance under extreme conditions. It is well known that properties of polymers start to deteriorate at temperatures above that of glass transition. As for temperature-dependent thermal conductivity, experimental studies conducted on epoxy resin above the glass transition temperature but below the thermal decomposition temperature [97,98] showed a linear increase in the thermal conductivity with temperature until 177 °C. No reported experimental measurements for thermal conductivity have been found at temperatures above 177 °C. As for the specific heat, polymers do exhibit a nonlinear increase in specific heat prior to the start of thermal decomposition (i.e., ~300 °C for epoxy resins) [99].

The thermal decomposition (pyrolysis) of polymers consists of complex chemical reactions and the formation of new material phases, a solid pyrolytic phase, and pores filled with pyrolysis gases. These processes lead to drastic reductions in the polymer’s thermal conductivity and specific heat with temperature. As a result, significant reductions in the transverse thermal conductivity and specific heat of the polymer matrix composites are predicted [100].

Moreover, the mass loss of a polymer is strongly dependent on the heating rate. As the heating rate increases, the mass loss decreases and the temperature, at which the polymer is fully decomposed, increases. Thermal gravimetric analysis (TGA) experimental data show that the mass loss of carbon fiber-reinforced polymer (CFRP) composites under a heating rate of 50 °C/min is about 17% lower than that under a heating rate of 10 °C/min in nitrogen at 400 °C, and the difference continues to increase as the temperature increases [101]. In addition, the temperature at which the polymer is fully decomposed also increases with the increasing heating rates. For example, under a heating rate of 1 °C/min, the temperature at which the epoxy resin is fully decomposed is about 650 °C, whereas the temperature increases to about 800 °C when the heating rate is increased to 20 °C/min [19]. For instance, under lightning strike conditions, the temperature of the material rises to several thousand degrees within a few microseconds. Therefore, the resin decomposition rate is expected to be significantly lower than those obtained in TGA tests, and the temperature at which the resin is fully decomposed is expected to be much higher than 800 °C.

As for the char, it is a highly porous solid carbonaceous residue and is a byproduct of the pyrolytic thermal decomposition. Polymers, when exposed to high temperatures, are decomposed by three major mechanisms; these are random chain scission, chain-end scission, and chain stripping [102,103]. Different classes of polymers exhibit different reaction mechanisms, and the overall char content that is produced differs.

Mechanical properties, such as stiffness, strength, and viscoelasticity, also exhibit temperature sensitivity, affecting the material’s response to mechanical loads induced by the thermal and electromagnetic effects of a lightning strike. Experimental data show that the elastic modulus of epoxy exhibits a steep decrease as the temperature approaches its glass transition temperature [104]. Poisson’s ratio of epoxy also exhibits a strong dependence on the temperature. Previous experimental studies reported an increase in Poisson’s ratio up to a value of 0.5 at the glass transition temperature [105,106,107]. A detailed micromechanics-based study on the temperature-dependent elastic properties of CFRP composites at high temperatures was reported in [108].

In summary, understanding and incorporating temperature-dependent properties into the predictive modeling of lightning strikes on composites is vital for capturing the intricate interplay between electrical, thermal, and mechanical phenomena. Enhanced models that consider these properties can facilitate more reliable predictions of the behavior and performance of composite materials under lightning strike conditions, ultimately contributing to the advancement of safety and durability in various industries.

## 5. Lighting Electric Arc Plasma Modeling

There are two main purposes of modeling the lightning electric arc. The first purpose is to acquire the accurate loading condition, including the heat flux, current density, plasma pressure, and electromagnetic force impinging on the surface of the CFRP composite material as well as the arc radius expansion. Note that in many existing lightning strike modeling studies, the applied heat flux and current density are based on assumed profiles and assumed arc radii [21,22,37,38,39,40,41]. The second purpose is to understand the interaction between the electric arc and the material behavior of the composite specimen, for example, how do the particles vaporized from the composite material during the lightning strike affect the electric arc characteristics [109]. Overall, studies on the modeling of lightning electric arc plasma are still scarce to date, primarily due to the challenges in the numerical implementation and the associated expensive computational run time (some can take 50 days [47]). Therefore, although modeling the lightning electric arc plasma is critical for determining the accurate loading conditions and studying the complex interaction between the composite and the electric arc, the trade-off between the computational cost and the improvement in the accuracy of the prediction remains a significant issue. For example, studies have shown that when using traditionally assumed arc radius loading, the moderate and severe lightning damages were underpredicted by 61% and 2.75%, respectively, while using the arc radius loading predicted from a plasma model, the moderate lightning damage was underpredicted by 50% and the severe lightning damage was overpredicted by 129.5% [44,110]. Therefore, for engineering applications, unless the computational cost is significantly reduced and the predictive accuracy is substantially improved, the necessity of creating a plasma model to determine the accuracy loading condition is questionable. Currently, most of the modeling studies have focused only on the material response and the damage of composites under prescribed lightning strike loading conditions. Nevertheless, the efforts on lightning strike plasma modeling will be concisely reviewed here to inspire future innovations in numerical implementations (e.g., physics-constrained neural-network deep learning). 

The review will be presented from two perspectives: (1) a lightning electric arc plasma–magnetohydrodynamic (MHD) model and (2) a lightning plasma–composite damage coupled model. The review will emphasize the numerical challenges and future recommendations.

### 5.1. Lightning Electric Arc Plasma–Magnetohydrodynamic Model

**Figure 4:**
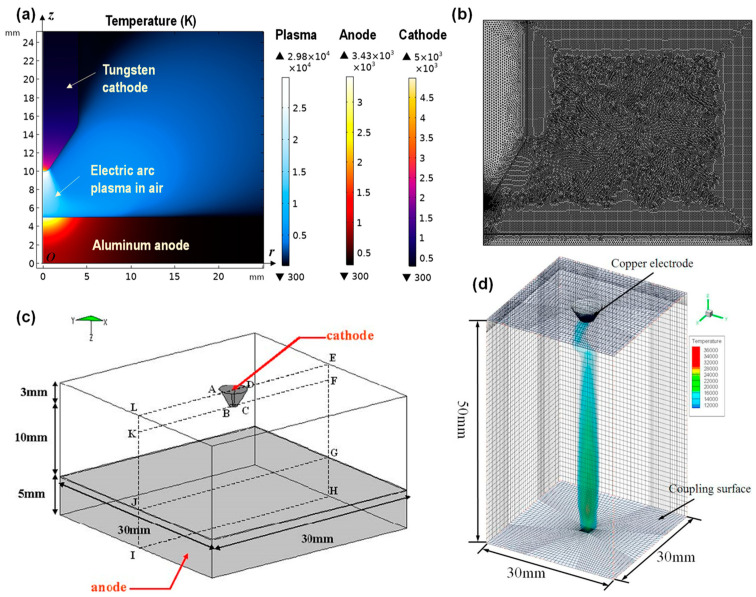
Examples of computational setups for modeling the lightning strike electric arc plasma using the MHD method for predicting the plasma temperature, heat flux, current density, arc pressure, etc.: (**a**) 2D axis-symmetric model [109], (**b**) example of the mesh used for the 2D axis-symmetric plasma model [44], (**c**) 3D lightning strike electric arc plasma model [111], and (**d**) 3D predictions of the plasma temperature [42].

This approach currently used to model the lightning strike electric arc plasma follows the same magnetohydrodynamic (MHD) method used to model the electric arc for welding applications. The differences between the lightning strike electric arc and the welding electric arc are as follows: (1) the lightning strike electric arc is produced in the air, whereas the welding arc is produced mostly in the inert gas environment (e.g., argon); and (2) the electric current for lightning strike electric arc consists of four waveforms, and the peak current can reach 200 kA, whereas the electric current for the welding arc is normally a constant current ranging from 100 A to 800 A. The MHD-governing equations include Maxwell’s equations, the Navier–Stokes equation, the heat transfer energy balance equation, and the conservation equation of electric charge. These equations are classical and widely known to the research community and are not reviewed here for brevity. The typical computational domain consists of a cathode, anode, and the air (or inert gas for a welding arc). Figure 4 shows a few examples of the computational problem setup for the MHD model. The model is often simplified in an axis-symmetric configuration to reduce the computational cost (see Figure 4a,b). Here, one needs to be careful with using the axis-symmetric configuration. Such a configuration is valid only for isotropic materials (e.g., copper, aluminum, steel). When it comes to laminated composite materials, since they are anisotropic, the axis-symmetric configuration is invalid regardless of modeling a unidirectional or multidirectional laminate. Thus, a three-dimensional (3D) model needs to be created (see Figure 4c,d). A quasi-isotropic laminate may be crudely assumed to be in the axis-symmetric configuration. However, sufficient numerical evidence, such as a comparison between the predictions using a 2D axis-symmetric configuration and the predictions using a 3D model still needs to be provided to prove the accuracy of the assumption. 

Researchers have used the MHD lightning strike electric arc plasma model not only to determine the loading conditions from the electric arc to the surface of the CFRP composites [44,46,47,111], but also to study the effect of testing configurations on the characteristics of the electric arc and the resulting damage in the anode material (e.g., metallic materials, CFRP composites) [8,44,51,112]. The latter studies have demonstrated that the arc gap (i.e., the distance between the cathode and anode) plays a significant role in determining the loading condition in the electric arc. For example, it was shown that the heat flux decreased by 80% when the arc gap was increased from 1 mm to 10 mm [51]. Some other studies have also investigated the effect of the size of the electrode. Moreover, the choice of using an indirect electrode or direct electrode for the simulated lightning strike test is still in debate. Studies have used the MHD model and investigated the effect of the metal vapor produced during the vaporization of the initiation wire used in the indirect electrode setup. It was found that the metal vapor significantly changes the net emission coefficient and the electrical and thermal conductivities of the electric arc, which results in significant changes in the damage of the tested material [112,113,114]. 

Current challenges in modeling the lightning strike electric arc plasma using the MHD method primarily include the following aspects:

(1) Difficulty in obtaining material properties for the electric arc. These properties include mass density, net emission coefficient, electrical and thermal transport properties, and viscosity, which are all temperature dependent. Although these properties have been predicted using theoretical methods (e.g., thermodynamic), predictions are not quite consistent among different research groups. More importantly, experimental data are severely lacking, especially for transport properties at high temperatures beyond 15,000 K (the plasma temperature can go above 20,000 K) [48,49] as well as the net emission coefficient of the plasma in air [115] due to the difficulty in experimental instrumentation. Future research is needed to develop instrumentation techniques to enable the measurement of these properties that are critical to the accuracy of the MHD plasma model.
(2) Numerical convergence and computational cost. Most MHD models for welding electric arcs use a constant electric current. However, for lightning strikes, especially for electric current waveforms A, B, and D, the electric current quickly rises to kiloamperes within only a few tens or hundreds of microseconds. This poses significant challenges to the numerical convergence as the corresponding material properties experience dramatic changes within a short duration, which makes the problem highly nonlinear. The time increment needs to be sufficiently small to capture the changes in the plasma temperature, velocity, and other observables as the lightning current drastically increases. The mesh size also needs to be sufficiently small to accommodate the extremely short time increment to avoid mesh-dependent solutions. It has been reported that the run time for a complete waveform B MHD simulation using COMSOL Multiphysics (i.e., a general-purpose commercial finite element software) takes about 70 days even with parallel computing using high-performance computing [47]. To solve this issue, the same researchers have adopted a scaling approach based on the similitude theory and successfully reduced the run time to less than 10 days. Although the run time has been significantly reduced, it is still computationally cost-prohibitive for many practical engineering applications. Due to the issues with numerical convergence, many studies have focused only on a single or a few of the four lightning current waveform components, such as modeling waveform C (i.e., constant current) only [10,112,113,114], waveforms B [44,45,47], or waveforms A and C [42,116]. To the authors’ knowledge, the plasma model for four complete lightning waveforms is not available to date. Therefore, there is a need in the future to develop a plasma model for all four lightning waveform components and examine how the predictions are different from those predicted using only one or a few waveforms. Moreover, novel numerical algorithms need to be developed in the future to speed up the computation. One potential solution is to create cost-effective surrogate models using machine learning methods.
(3) Difficulty in representing a true lightning strike discharge in the air. The current MHD models for lightning strike electric arc are mostly created based on laboratory lightning strike test setups. The arc gap between the cathode and anode is limited to only a few millimeters. As discussed previously, the arc gap has a significant impact on the lightning strike loading conditions as well as the arc radius and its expansion. In the field, a lightning strike arc has a length of about 4 km, and the radius can reach 1 m [117]. However, increasing the arc gap in the MHD model will significantly increase the difficulty in achieving numerical convergence and will greatly increase the computational cost. The convergence will start to become extremely difficult when the arc gap increases to 20 cm [47,51]. Although the first author and third author modeled a 4 km long electric arc in their prior works [118,119], the models solved only the electric field and ignored the magnetic field and the flow field. In addition, the actual environment, such as the humidity, environment temperature, dust, wind, and operation conditions (e.g., the flight speed of an aircraft and speed of a blade for a wind turbine) can all have influences on the characteristics of the electric arc plasma produced in the air. Therefore, future research is recommended to improve the fidelity of the plasma model to understand the various factors that are affecting the lightning strike electric arc characteristics and the effect on the composite structures.

### 5.2. Lightning Plasma–Composite Damage Coupled Model

Recall that the purpose of modeling the lightning strike electric arc plasma is to determine the loading conditions on the lightning-attached surface of the CFRP composites. After that, the determined loading conditions are used in the lightning strike composite damage model for the damage prediction. There are typically two approaches for achieving this: one is to sequentially couple the lightning composite damage model from the lightning electric arc plasma model. For example, Millen et al., [44,45] predicted the lightning loading conditions using a COMSOL MHD lightning strike model for a Waveform B current first. Then, the output of the plasma model, i.e., the predicted loading conditions were transferred to a separate material damage simulation as model input to predict the material damage under lightning strike loading. The mesh and loading boundary conditions of the composite material were automatically generated based on the predicted output of the plasma model. The other approach is to synchronously couple the lightning plasma model with a lightning thermal damage model. This approach improves the fidelity of the model as it captures the in situ interaction between the plasma and the composite material, which is more computationally challenging. Studies using this approach are limited. For example, Chen et al. [42] developed a loosely coupled ANSYS and Fluent model to predict lightning strike damage in a composite material. They developed an interpolation subroutine to exchange the information between the ANSYS thermal damage model and the Fluent plasma model. Specifically, the interpolation subroutine transfers the heat flux, electric current, and force from the plasma model to the thermal damage model and at the same time transfers the temperature, electric potential, and displacement from the thermal damage model to the plasma model. 

In addition to the challenges discussed in the previous section related to the lightning strike electric arc plasma model itself, there are other challenges that researchers are facing in coupling the plasma model with the thermal damage model. A few important challenges that future work is recommended to address include (1) developing strategies to effectively exchange information between the plasma model and the thermal damage model while maintaining the numerical accuracy and numerical convergence of both models, (2) developing strategies to handle the moving boundary conditions in both the plasma model and the thermal damage model caused by the progressive material removal (i.e., surface resin vaporization and fiber sublimation), and (3) develop methods to account for the effect of particles vaporized from the material during lightning strike on the plasma characteristics.

Significant research progress has been made on the modeling of the lightning strike material and damage responses of CFRP composites since Ogasawara et al. [19] first presented an electric–thermal coupled model for simulating the lightning strike response of CFRP composites in the year of 2010. Progress has been mainly achieved in the following aspects. (i) More physically representative lightning strike loading conditions have been used in the model to account for the electric arc radius and the arc expansion, as well as the spatial variation in the heat flux and current density within the electric arc. These loading conditions are either assumed or determined based on experimental data from welding arcs or using a separate/coupled MHD model, which have significantly improved the fidelity of the lightning strike damage model and the accuracy of the prediction [110]. (ii) Temperature-dependent material properties, including density, electrical conductivity, thermal conductivity, and specific heat have been used in the electric–thermal coupled model to understand the effect of material degradation on the thermal damage [120,121]. (iii) The pyrolytic behavior of the polymer matrix at elevated temperatures has been incorporated into the electric–thermal coupled model to account for the resin decomposition caused by Joule heating [38,63,122]. (iv) The mechanical forces caused by the lightning strike, such as the acoustic shock wave and the arc pressure, have been either separately modeled or coupled to the electric–thermal model [22,37,59]. (v) The effect of the protection layer, such as the expanded copper mesh on the lightning strike damage response of the CFRP composite substrate has been modeled. (vi) Delamination caused during the lightning strike impact has been modeled using a cohesive zone method with mechanical interfacial properties (e.g., interface stiffness, strength, and fracture energy) [123,124]. (vii) Ablation caused due to the resin vaporization and carbon fiber sublimation has been modeled using element deletion methods [39,40,41]. (viii) Thermal expansion has been considered with the heating rate- and strain rate-dependent material properties to understand the thermal strains caused due to the rapid heating and its constraints relative to the experimental boundary conditions [58,125]. Also, some other models have introduced failure criteria (e.g., Hashin) to predict the fiber and matrix damage initiation and the damage propagation along with the thermal damage caused by a lightning strike [37,59]. 

A detailed review of the problem formulations and modeling methods of the lightning strike response of CFRP composites can be found in [57,110], and hence, is not re-reviewed here for brevity. Below, we will point out only the primary challenges related to lightning strike damage modeling and provide some future recommendations:

The first challenge is due to the lack of temperature-dependent material data, which include density, electrical and thermal conductivities, specific heat and mechanical properties from room temperature to the sublimation point of carbon fiber (i.e., around 3000 °C), and the lack of resin decomposition kinetics at extreme high heating rate- and strain rate-dependent thermal expansion coefficients. These material parameters are extremely vital to the accuracy of the model because the predictions (e.g., temperature) are highly sensitive to them. However, acquiring these material data is difficult due to the limitations in the experimental instrumentation, especially at high temperatures. The material data currently used in models reported by different research groups showed a large inconsistency. A summary of this inconsistency is presented in a recent review by Millen [110]. Future research on this topic is recommended to look into (i) the development of experimental techniques to enable the determination of these material data, especially at high temperatures and at extreme high heating rate; (ii) the development of models, such as models using molecular dynamics and/or thermodynamics to predict the material properties at high temperatures; and (iii) quantifying the stochastic lightning strike damage response by considering the uncertainty in the material parameters [126].

The second challenge is related to the difficulty in modeling the evolution of all damage modes concurrently. Although the progressive damage modeling methods of CFRP composites under conventional mechanical loadings are well established by including the failure criteria (e.g., Hashin, Puck, NU-Daniel) and material degradation functions [127], these methods are unable to capture the complex damage modes in CFRP composites subjected to lightning strike. Multiphysics loading conditions involve the material loss due to resin vaporization and carbon sublimation, charring, delamination due to the loss of resin between adjacent layers, fiber breakage caused by interlaminar explosion and possible potential internal arcing, and dielectric breakdown (which was reported as the main cause of the edge glow effect in lightning strike tests [128]), in addition to those traditional mechanical damage failure modes. Currently, most lightning strike modeling studies model the different damage modes separately. For example, some studies focused on the electric–thermal damage [19,23,40], and some focused on the mechanical damage caused by shock waves [58,61], while some focused on thermal expansion [57]. One study proposed an innovative methodology to simplify the lightning strike multiphysics phenomenon into a purely mechanical problem by reproducing the mechanical load produced by the lightning strike explosion pressure [129]. This offers an alternative surrogate modeling strategy. Future research will need to investigate the coupling effects among the different damage modes and understand the critical material parameters that are dictating each damage mode. For example, questions of “what is the ablation rate of carbon fiber?” and “how does the pressure build up in between adjacent plies due to the release of pyrolysis gases and how does this pressure lead to fiber breakage and delamination?” need to be answered. Advanced models will need to be developed to account for all concurrent damage modes caused by a lightning strike and elucidate the importance of considering all concurrent damage modes vs. considering a few single damage modes (e.g., delamination and resin vaporization).

The third challenge is to consider all four sequential lightning waveform currents in a single model. It has been experimentally proved that the damage of the CFRP composites is much more significant when a sequence of four lightning waveform currents is applied compared to when only a single waveform (e.g., waveform A) is applied (see discussions in Section 2). To model a sequence of four lightning waveforms, the challenge is mainly caused by the large difference in the time scale and dynamics of different waveforms and the difficulty in the highly coupled numerical implementation. For waveform A, the current rises to a few hundred kiloamperes within a few microseconds and decays to a few hundred amperes within tens of microseconds. To capture the transient temperature and the resulting thermal damage caused by this rapidly changing electric current, the time increment needs to be sufficiently small. This demands the mesh to be sufficiently small to accommodate the time increment and avoid mesh-dependent predictions. For waveform C, the lightning current stays at about a few hundred amperes for 0.5 to 1 s. Therefore, if the same mesh and time increment used for waveform A are used for waveform C, the simulation is likely to result in unaffordable computational time. To overcome this challenge, future research is recommended to develop novel numerical algorithms or modeling methods to accelerate the numerical computation, such as by reducing the frequency of coupling between the electric field and the temperature field from every time increment to every a few time increments, developing a sub-model of the CFRP composite with an extremely fine mesh for the lightning-attached region and incorporate the sub-model into the global full CFRP model with a much coarser mesh, and/or using different mesh configurations for different waveforms and map the results from the previous mesh configuration to the subsequent mesh configuration as predefined initial conditions. Additionally, machine learning methods can be used to develop surrogate models to significantly reduce the computational run time while achieving equivalent accuracy.

Overall, despite significant progress having been made on the modeling of lightning strike damage response of CFRP composites, improving the model fidelity is still challenging as described above. In parallel with the development of more advanced models, model validation also needs to be improved to provide more confidence on the validity and effectiveness of models. Currently, validations of the lightning strike models have been mostly achieved by comparing the predictions of the extent of delamination (depth and area) and surface vaporization. Future research is recommended to further validate models by also comparing against the extent of the charred region, the temperature history on the back side of the CFRP composite, the displacement history of the CFRP composite, the exact mass loss of the composite, and the extents of the fiber breakage and matrix cracking.

## 6. Conclusions

This paper presents a thorough review on the present status of lightning strike testing and modeling for composite materials, which emphasized the methodologies for enabling a robust and high-fidelity predictive capability for lightning strike damage assessments. Although significant progress has been made over the past decade, many challenges still exist in both laboratory testing and predictive modeling, including generating arcs that are more representative of actual lightning arcs, probing the electric current density and heat flux within a lightning arc column to acquire more accurate lightning loading conditions and for plasma model validations, establishing a universal and consistent test setup for electrodes and grounding conditions, validating damage models against transient damage evolutions during lightning impact, determining and integrating more accurate temperature- and strain-dependent material parameters, and developing predictive electric arc plasma and material damage models considering combined lightning waveforms. Recommendations for addressing these challenges have been presented in this review, which point out potential future directions in this field toward an enhanced and more reliable predictive capability for lightning strike damage assessments and the development of damage mitigation strategies.

## Figures and Tables

**Table 1:** Comparison of laboratory lightning strike characteristics with actual lightning strikes.

	Actual Lightning Strike	Laboratory Electric Arc
Peak current	200 kA (±10%)	200 kA (±10%)
Peak power	~10^11^ W/m	~10^11^ W/m
Action integral	2 × 10^6^ A^2^·s (±20%)	2 × 10^6^ A^2^·s (±20%)
Discharge mechanism	Breakdown of the air	Breakdown of the air
Arc length	4000 m	3~10 mm
Arc radius	1 m	A few centimeters
Peak voltage	In the order of tens of kilovolts	10~120 million volts
Acoustic shock wave	~400 Pa	2 Pa (recorded with microphones at 1.8 m in a 5 kA experiment) [34]

**Table 2:** Electrical conductivity of unidirectional CFRP composites at room temperature and comparisons between results reported in different studies.

Models	Electrical Conductivity (S/mm)		
	*σ*_1_	*σ*_2_	*σ*_3_
Wang (AS4/8552) [42]	33.8	1.690 × 10^−3^	2.704 × 10^−4^
Ogasawara (IM600/133) [19]	29.3	0.787 × 10^−3^	7.940 × 10^−7^
Abdelal (IM600/133) [21]	35.97	1.145 × 10^−3^	3.876 × 10^−6^
Muñoz (G0986/RTM6-2) [22]	14.631	Not available	2.700 × 10^−3^
Liu (Not specified) [23]	34.6	1.220 × 10^−3^	3.240 × 10^−6^
Kawakami (T700/2510) [95]	23.09	8.000 × 10^−3^	1.1236 × 10^−4^
Kawakami (T800/3900) [95]	16.58	1.028807 × 10^−3^	8.4034 × 10^−5^
Kawakami (IM7/977-3) [95]	39.68	1.964637 × 10^−3^	3.22581 × 10^−4^

## Data Availability

Not applicable.
